# Prediction of ABX_3_ Perovskite Formation Energy Using Machine Learning

**DOI:** 10.3390/ma18132927

**Published:** 2025-06-20

**Authors:** Ziliang Deng, Kailing Fang, Chong Guo, Zhichao Gong, Haojie Yue, Huacheng Zhang, Kang Li, Kun Guo, Zhiyong Liu, Bing Xie, Jinshan Lu, Kui Yao, Francis Eng Hock Tay

**Affiliations:** 1 School of Power and Energy, Nanchang Hangkong University, Nanchang 330063, China; dengzleon@163.com (Z.D.); kalen_fang@163.com (K.F.); cguo198131@163.com (C.G.); bqibao2000@163.com (Z.G.); b1924938193@163.com (H.Y.); zsviq946410@163.com (H.Z.); 16673823327@163.com (K.L.); zyliu@nchu.edu.cn (Z.L.); xieb@nchu.edu.cn (B.X.); jinshan.lu@nchu.edu.cn (J.L.); 2 Institute of Materials Research and Engineering, A*STAR (Agency for Science, Technology and Research), 2 Fusionopolis Way, Singapore 138634, Singapore; k-yao@imre.a-star.edu.sg; 3 Department of Mechanical Engineering, National University of Singapore, 9 Engineering Drive 1, Singapore 117575, Singapore; mpetayeh@nus.edu.sg

**Keywords:** ABX_3_ perovskites, perovskite solar cells, formation energy, machine learning

## Abstract

Materials with perovskite phases are widely used in solar cells and ferroelectric, piezoelectric, dielectric and superconducting devices due to their various notable functions. However, structural instability limits some compositions in forming robust perovskite phases for device applications. The analytical approach using the tolerance factor (t) can only guarantee prediction accuracy within a limited range, ascribed to its nature of overlooking the atomic interaction. Hence, here we establish a prediction model using formation energy as the target parameter for its reflection of the reaction of atoms and apply machine learning as the analysis method since it has been successfully employed in plenty of material property prediction studies. Machine learning employs statistical methodologies to identify correlative patterns within large-scale datasets, enabling accurate predictions with robust generalization. In this work, we built a model to predict the formation energy of ABX_3_ perovskite using machine learning and achieved a model with an R-squared value of 0.928 and a root mean square error of 0.301 eV/atom, validated by first-principles computations. In total, 75% of the values were correctly predicted within an error lower than 0.06. This work could contribute to accelerating the study of solving perovskites’ instability.

## 1. Introduction

Inorganic perovskites are widely used in solar cells and ferroelectric, piezoelectric, dielectric and superconducting devices due to their various notable functions [1,2]. However, researchers have found a significant concern that has prevented perovskites from being widely used in certain applications, which is their instability towards phase, light, and moisture. For instance, Liu et al. [3] found that all inorganic lead halide perovskites (ILHPs) exhibit excellent thermal stability compared to hybrid organic–inorganic perovskites. Yet the low structural stability against ambient conditions still limits their practical applications. Xiang et al. [4] pointed out that although the power conversion efficiency of inorganic perovskite-based solar cells has reached over 20%, inorganic perovskite materials still suffer from instability towards phase, light, moisture, etc. These instability issues seriously affect the device’s performance and pose as one of the major concerns [2,5]. Ouedraogo et al. studied the stability of all-inorganic perovskite solar cells and concluded that under the operating temperature on earth, the stability of inorganic lead halide perovskite materials remains the most crucial issue to overcome [6]. The biggest challenge in Wang et al.’s work in studying inorganic perovskites, especially CsPbI_3_, is phase stability at room temperature (RT) [7]. The structural stability issue also exists in the oxide perovskite phase for ferroelectric, piezoelectric and high dielectric applications, such as in Pb(Zn,Nb)O_3_ and Pb(Zn,Nb)O_3_ systems [8,9]. Therefore, future work should focus on solving the stability issue for the broader development of inorganic perovskites.

Some studies tried to solve this through chemical modification methods such as doping and indeed made some progress. For example, Li et al. [10] used large-radius cations in the CsPbI_3_ perovskite to replace Cs+, significantly improving the film morphology as well as the phase stability. Other methods include improving the material processing method [11].

Nevertheless, these approaches require tedious experimental exploration and resources in the lab but have little appeal. Hence, another train of thought is to use some parameter as a reflection of the stability of the target material, offering an insight to researchers before they invest effort. The tolerance factor (*t*) has become the most extensive research object. In the ABX_3_-type perovskite structure (covering ABO_3_), the tolerance factor (*t*) proposed by Goldschmidt can be expressed as follows:

(1) $$t=\frac{r_{A}+r_{X}}{\sqrt{2(r_{B}+r_{X})}}$$

where *r_A_*, *r_B_* and *r_X_* are the ionic radii on A-site, B-site and X-site, respectively. In the case of mixed occupancies, it is the average ionic radius at the designated position. Yan et al. [12] used the tolerance factor *t* in their work to conjecture the distortion level in the lattice of BT-based perovskites to obtain a reflection of their stability performance. In the research of Coondoo et al. [13], Yan et al. [12], and Yu et al. [8], *t* also played a similar role.

The aforementioned tolerance factor *t* can be used to estimate whether a stable perovskite structure is expected to be formed dependent on the composition [4]; there exists some situations that are beyond the prediction ability of *t*. Fundamentally, *t* is a geometric approach based on the assumption of a hard sphere model, which becomes less valid when it comes to iodide anions with their lower electronegativity of the heavier halides and greater chemical softness [14]. This is confirmed in the work of Travis et al., where they applied a range of stabilities for hybrid iodide perovskites, roughly 0.8 ≤ *t* ≤ 1, as suggested by Cheetham and co-workers [15,16], which is very similar to that found for oxides and fluorides, but it turns out that this criterion is not that satisfying due to the low accuracy. Hence, it can be concluded that *t* may be a necessary condition for perovskite formation, but not a sufficient condition, and the traditional approach that works reasonably effectively for fluoride and oxide compounds cannot be used to explain the known structures of the inorganic ABI_3_ compounds [14]. Besides the tolerance factor t, there is another parameter, the octahedral factor *μ*, which is the ratio of the radii of the B-site cation and the X-site anion, often being employed alongside t to evaluate the stability of perovskites. Zhao and co-workers [17] used both *μ* and *t* in their work, finding out that in a complex perovskite system where there exist mixed ionic and covalent bonding and multinary features, it is challenging to assign realistic *t* and *μ* for evaluating crystallographic stability. These results mean that, although *t* or *μ* can provide a qualitative range for the formability of perovskite, it is not a good stability descriptor, i.e., quantitative correlation with stability [18]. In addition to this, some researchers tried another path by carrying out a mathematical process on *t* and *μ* and creating new parameters. For example, Sun and co-workers [18] created (*μ* + *t*)*^η^* and improved the prediction with an accuracy of around 90% by bringing the atomic packing fraction (APF) into consideration for correcting the error, which is much better than using *t* or *μ* alone.

The studies mentioned above have demonstrated research efforts in improving the generalization ability of various relevant parameters to represent the stability of perovskite structures, yet all these approaches have a commonality of overlooking the correlation between the ions since the theory of *t* and *μ* assumes the maximum anion contact with cations at the very first moment, which means they can only improve the prediction of stability in a limited way. Therefore, we herein address a more effective parameter that can not only recapitulate the interaction of the atoms but also is capable of generalization. We choose the formation energy (E_form_) because it can reflect the chemical interactions between elements [19] and represent the bonding strength of atoms within a material system. It correlates with many thermodynamic and kinetic properties, and thus the stability [20] and synthesizability [21] of a compound [22]. Furthermore, the states and distribution of defects are determined by the formation energy of the configuration and the entropy of the system. Thus, this makes detailed calculations regarding the formation energies of different defect configurations and entropies of various systems essential for defect configuration design [23].

It is also noteworthy that some researchers already utilized E_form_ as a basis for determining whether a compound is stable and for comparing the stability of different compounds in their work [24]. For example, Xiang et al. [4] proved that the increasing intrinsic formation energies of vacancies is a way to decrease their concentration and make perovskites more stable against external factors, as suggested by Saidaminov et al. [25]. Zhou et al. [26] pointed out that the substitution of A-, B-, and X-site ions with other corresponding ions can tune the formation energies, resulting in more stable compounds. Wang and co-workers [7] addressed this in the work of Hu et al. [27], where CsPb_0.6_Sn_0.4_I_3_ exhibited higher oxidation stability compared with its hybrid counterpart, as the smaller Cs^+^ leads to stronger antibonding of Sn 5s with I 5p and thus the smaller formation energy of Sn defects in inorganic perovskites. Liu et al. calculated E_form_ before deciding which site in their targeted perovskite system to dope to ensure their outcome compound was stable [3]. From another perspective, E_form_ could also be applied to confirm that compounds are not stable. For example, Sutton et al. [28] calculated the formation energies concerning the precursors of CsI and PbI_2_, finding out that the compounds are unstable. In summary, E_form_ plays an essential role in evaluating perovskite stabilities [29,30].

**Figure 1:**
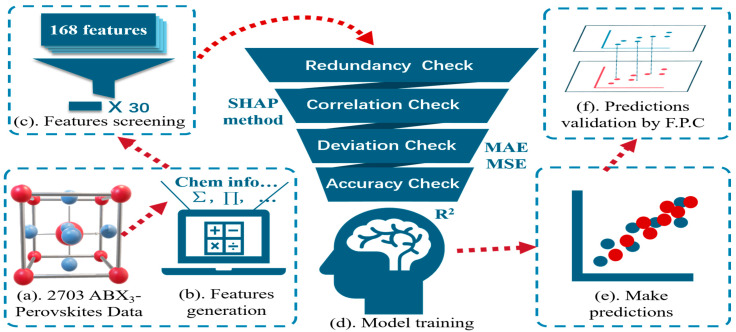
Workflow of the ML strategy to predict the formation energy of ABX_3_ perovskites. (**a**) Dataset collection. (**b**) Feature generation. (**c**) Feature screening by Pearson correlation coefficient. (**d**) Model training. (**e**) Make predictions with trained model. (**f**) Validate prediction by comparing with first-principles computations.

However, despite the fact that E_form_ is capable of evaluating perovskite stabilities, it is not easy to obtain; usually, researchers use first-principles computation (FPC) to calculate it, with a huge workload. This often makes the result deviate from the essence of prediction for saving unnecessary work input. We will use a machine learning (ML) tool, which is a big data-driven approach covering predictive analytics, clustering, relationship mining, and anomaly detection [31,32]. Usually, building an ML model should include the following steps: data collection, feature generation and screening, and model training and evaluation, with the workflow shown in Figure 1. In general, the underlying logic of ML is essentially a statistical method that finds correlations between massive features of certain systematic materials and their properties to obtain a mutual correlation. Attributed to this, ML can reach accurate predictions and possesses natural generalization capabilities. More details about ML will be discussed in the following Section 2.

Motivated by these considerations, we believe that ABX_3_ perovskites and the instability issues in certain applications are very worthy of studying [33,34,35,36,37,38]. In addition, E_form_ as an indicator of stability also has lots of application potential [30,39]. Starting from the instability problem of perovskite in certain applications, this work demonstrates the limitations of the existing tolerance factor and octahedral factor, as well as the rationality of using the formation energy as a stability indicator. We selected machine learning as the main research method, and through the optimization training of multiple model algorithms, we found a balance between computing power consumption, accuracy and error, and combined this with the SHAP method to analyze the descriptor to improve the interpretability of the model. Finally, FPC calculation was used to produce an intuitive display of the application scenarios of the model.

## 2. Methodology

As the fourth paradigm of materials science [31], ML is essentially a method of materials informatics [32,40]. Materials informatics is defined as the implementation of data science in the problems inherent in materials science to accelerate the design and discovery of materials [41]. After years of development, the field of materials science has accumulated massive databases, which contain huge value as there are inherent correlations between them [42,43]. From the perspective of statistics and computational science, these correlations can be mined to accelerate the discovery and performance optimization of new materials [44,45,46]. This concept echoes the Materials Genome Initiative (MGI), and together they promote the progress of materials science [32,47,48,49].

Although ML has become more and more mature as a result of the research of many scholars in recent years, we still need to point out that due to the working principle of ML methods, they still have some limitations [50,51,52]. We summarize these into three aspects: first, data dependence and generalization ability issues, which are caused by the size of the dataset [53] and experimental data noise or annotation bias [51]; second, the black box effect and interpretability limitations, primarily due to the inherent lack of transparency in their decision-making processes [53]; third, challenges in practical applications, including, but not limited to, computational resource constraints, deployment scalability issues, and robustness deficiencies in real-world environments [54,55].

In our work, we aim to address the limitations of the ML methods mentioned above by introducing SHAP feature analysis to enhance the interpretability of the model (Section 3.3) and combining it with FPC calculation to intuitively reflect the model’s application scenarios in practice (Section 3.4).

### 2.1. Dataset Establishment

The dataset is vital for the model performance, as the data quality could directly affect the model training. Our data includes information on the material’s crystal structure, elemental composition, physical properties, chemical properties, and synthesis methods; this information can be found in published research papers and material databases in the public domain, such as the Inorganic Crystal Structure Database (ICSD) [56], Materials Project [57], Open Quantum Materials Database (OQMD) [58], and AiiD [59]. They can be extracted from open source software libraries, for example, SciKit-learn [60], XGBoost [61], Keras [62], Magpie [63], and Pymatgen [64]. These datasets collect data from various research experiments and FPC. Thus, they have different ways of expressing data and different scales of capacity, which require us to preprocess the material data, including removing null values and outliers, to make the data distribution more reasonable and uniform so as to improve the predictive ability of the model. All the software and packages for this work are listed in Table S3, Supplementary Materials.

### 2.2. Feature Generation

After a decent dataset is established, feature engineering is required, i.e., generating and screening features that are key to the target attributes. Usually, corresponding feature descriptors, such as ionic radius, atomic number, and element electronegativity, can be generated based on the material’s structural information and chemical composition to expand the dataset. Based on the feature selection method, the corresponding important features are screened to train the ML model. The whole process may involve operations such as feature selection, conversion, and dimensionality reduction. After this process, the dataset is capable of being processed by ML models.

### 2.3. Model Selection

We start the model selection to obtain the most suitable algorithm model or model combination to train the processed dataset. There are plenty of algorithms for selection like linear regression (LR), support vector regression (SVR), neural network multilayer perceptron (MLP), decision tree regression (DTR), random forest (RF), and extreme gradient boosting (XGBoost). The algorithm model selection depends on the characteristics of the data, the complexity of the problem, and the research objectives. Hence, we need to implement algorithm models on our dataset, which is randomly divided into two portions by a certain proportion based on capacity, named the training set and the test set. The test set outcome is used for comparison with the training set outcome for the evaluation and cross-validation of the generalization ability and prediction accuracy of the model. To determine the accuracy, there are four common indicators, mean absolute error (MAE), mean square error (MSE), root mean square error (RMSE), and goodness of fit (R-squared, R^2^), where the smaller the values of the indicators MAE, MSE, and RMSE, and the closer R^2^ is to 1, the more reliable. The expressions are

(2) $$MAE=\frac{1}{N}\sum_{i=1}^{N}\left|y_{i}^{true}-y_{i}^{pred}\right|$$

(3) $$MSE=\frac{1}{N}\sum_{i=1}^{N}{\left(y_{i}^{true}-y_{i}^{pred}\right)}^{2}$$

(4) $$RMSE=\sqrt{\frac{1}{N}\sum_{i=1}^{N}{\left(y_{i}^{true}-y_{i}^{pred}\right)}^{2}}$$

(5) $$R^{2}=1-\frac{\sum_{i=1}^{N}{\left(y_{i}^{true}-y_{i}^{pred}\right)}^{2}}{\sum_{i=1}^{N}{\left(y_{i}^{true}-\bar{y_{i}^{true}}\right)}^{2}}$$

where $y_{i}^{true}$ I and $y_{i}^{pred}$ represent the experimentally measured and model-predicted formation energies, respectively, for each composition in our dataset.

### 2.4. Model Verification Means

By finishing the work above, we have acquired a well-functioning prediction model. To make it more persuasive, the models need to be verified with other methods according to the research subject. For example, He and co-workers [65] built a model with ML to predict the morphotropic phase boundary (MPB) to search for high-performance piezoelectric devices; they verified the results of the ML model by synthesizing compounds of the MPB region they predicted. In our work, synthesizing compounds would not be appropriate as E_form_ is not one of the properties that can be directly reflected by their detection. Therefore, we chose FPC to verify our model since E_form_ is usually calculated based on it. FPC is mainly based on density functional theory (DFT) [66]. The core idea of DFT is to express the ground-state energy of the system as a function of the electron density. The ground-state energy and electron distribution of the system can be obtained by solving the energy minimum value at a given electron density. This enables DFT to accurately describe the system without explicitly considering the wave function. Therefore, using FPC can naturally help us gain a reliable E_form_, and the work of Bartel et al. [67], using FPC to calculate the formation enthalpies, can help prove that.

## 3. Results and Discussion

### 3.1. Data Processing and Feature Screening

Our database is mainly based on the Materials Project database since it has a broad capability to realize a model with generalization ability through its open API port. With the screening conditions of the ABX_3_ structure, we obtained a total of 4358 compounds. After preprocessing, i.e., removing less helpful data, this was reduced to 2703 compounds. Except for the data for which we have do not directly differentiate the A, B, and X compositions of the compounds, for facilitating subsequent feature generation, we employed Python regular expressions to batch-select the most abundant X-site in the chemical formulas and, among the two elements left, assigned the element with a larger radius to the A-site and the other one to the B-site, leveraging the characteristics of perovskite compounds. Thereafter, we generated features of the 2703 compounds and created 168 features of each compound for future training.

**Figure 2:**
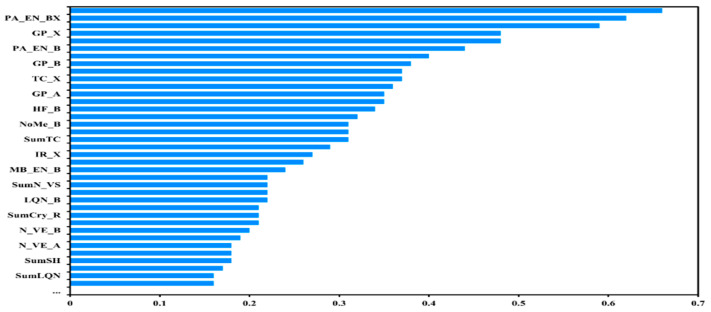
Importance ranking of features in describing the target feature.

With 168 features, there are bound to be redundant ones indicating similar properties, and therefore we used the Pearson correlation coefficient as a reflection of each feature against the others to remove those with a correlation greater than 0.8. In addition, the outlier data of *t* and *μ* and corresponding features were also removed, leaving 63 features for each compound. So far, our features have been simplified in the dimension of redundant features. We will further simplify the existing features based on the Pearson correlation coefficient from the perspective of the actual importance of each feature in describing the target feature. As shown in Figure 2, we chose the top 30 features for further model construction. Usually, from the aspects of computing power savings and efficiency considerations, the number of features should be reduced to the lowest possible number before model training; however, this method likely overlooks some features that have benefits to the model training. We looked into the probability distribution of the 30 remaining features, as shown in Figure S1 (Supplementary Materials). The result demonstrates a uniform distribution of the features within their respective ranges without notable anomalies.

### 3.2. Model Training and Performance Evaluation

**Figure 3:**
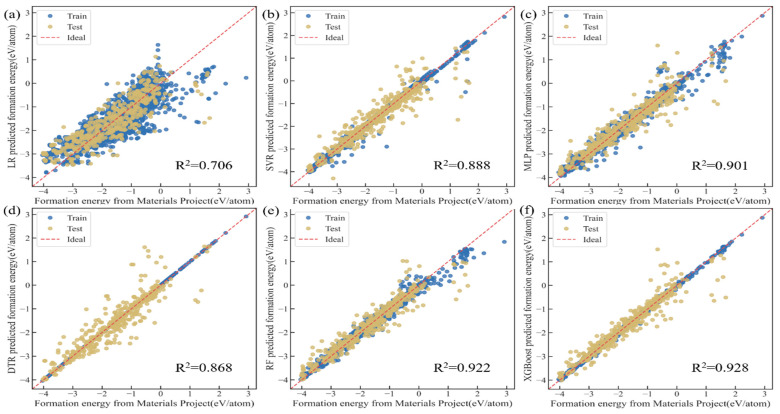
Thirty input features selected to compare the predicted value and the true value of various ML models: (**a**) LR; (**b**) SVR; (**c**) MLP; (**d**) DTR; (**e**) RF; (**f**) XGBoost.

We divide the dataset consisting of 30 screened features from previous work as input features plus one target feature (E_form_) into the training set and test set in a 4:1 ratio for model training and model verification, respectively. For model training, certain ML algorithms are selected, namely linear regression (LR), support vector regression (SVR), neural network multilayer perceptron (MLP), decision tree regression (DTR), random forest (RF), and extreme gradient boosting (XGBoost), to examine their matching ability with the 30 features for predicting E_form_. The specific results are shown in Figure 3.

As shown in Table 1, comparing the evaluation indicators of each model, the training effects of the models are ranked as follows: XGBoost, RF, MLP, SVR, DTR, and LR. Among them, the training effects of the RF and XGBoost models are relatively close. The goodness of fit R^2^ reaches 0.922 and 0.928, and the root mean square error is 0.313 and 0.301 eV/atom, respectively. In addition, to quantify the relative prediction error, we employed a normalized metric defined as the ratio of RMSE/average ($y_{i}^{pred}$). The XGBoost model still exhibited the best performance of 0.175. The model training achieved the ideal effect of higher accuracy and smaller deviation.

Figure 3a–f show the discreteness of the training set and test set of our model; the more the data distribution converges to the red diagonal line, the more accurate the model’s prediction results are. Figure 3e,f show a significantly better fit than the other models, which can be more intuitively illustrated by its R^2^. In addition, it is worth mentioning that the fit of the yellow points to the red diagonal line indicates that the model has excellent generalization ability to unknown data; in other words, it performs better in terms of predictive capacity.

### 3.3. Model Optimization and Feature Analysis

**Figure 4:**
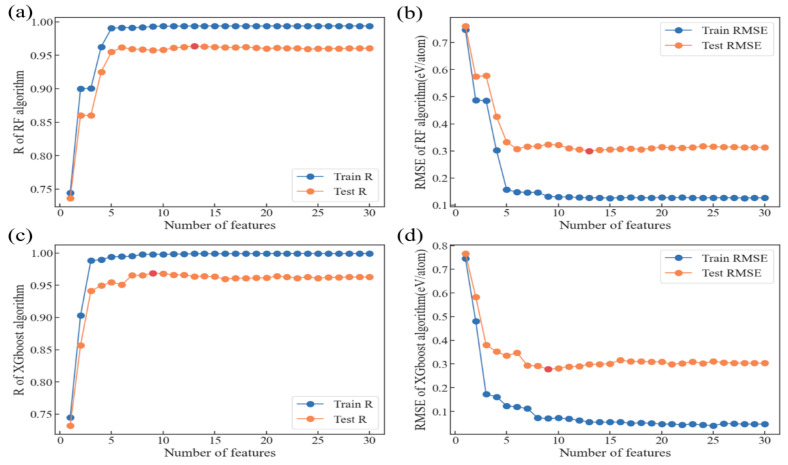
The relationship between the number of features and the accuracy and deviation of the model: (**a**) R of RF algorithm; (**b**) RMSE of RF algorithm; (**c**) R of XGBoost algorithm; (**d**) RMSE of XGBoost algorithm.

Our model is now functioning well. To further develop our model to a level of even better accuracy and to diminish the size of the model to save computing power, we use Shapley Additive Explanations (SHAP) to evaluate from another standard how many of the features we choose are actually contributing to the algorithms and if it is possible that there are still redundant features. By using the SHAP tool to sort the features of the two best-performing algorithm models, RF and XGBoost, according to their importance, as discussed above, we can obtain the relationship between the number of features and the accuracy and deviation of the two models, allowing us to remove some features that we kept for model selection. This helps us finally select the model, as shown in Figure 4.

As we assumed, the trends of R and RMSE against the number of features tend to stabilize or even decrease after reaching extreme values, indicating that there are indeed redundant features to be removed. We marked the lowest feature to realize the best R and the smallest RMSE for both models with red dots. Clearly, the RF model has the highest accuracy (R) and the smallest deviation (RMSE) in the test set at 13 features, while the XGBoost model only requires 9 features and even reaches a slightly higher R^2^. Therefore, we picked XGBoost as the ultimate model and further optimized it by discovering the nine best features required.

**Figure 5:**
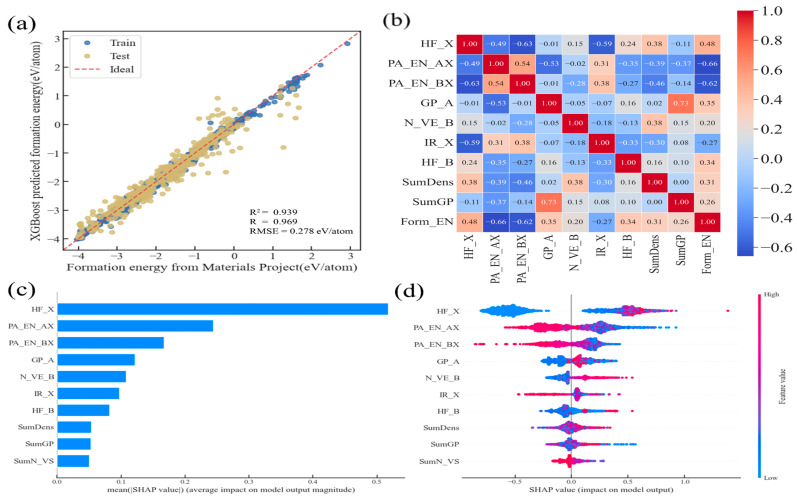
Results of retraining of the top 9 features on XGBoost model: (**a**) comparison of real formation energy and predicted value; (**b**) Pearson correlation heat map of the top 9 features and formation energy; (**c**) ranking of feature importance based on SHAP interpretation method; (**d**) feature analysis based on SHAP interpretation method.

We used SHAP to determine the nine key features with greater importance. As depicted in Figure 5c, the impact on E_form_ of each feature is shown in Figure 5d. Features with data points on the positive axis indicate a positive correlation with the target property; otherwise, they indicate a negative correlation. The higher the clustering of red points, the higher the correlation. With the nine features filtered, we retrained the XGBoost model results, as shown in Figure 5a,b. The model effectiveness of fit R^2^ has increased from the original 0.928 to 0.939, and the root mean square error (RMSE) has decreased from 0.301 eV/atom to 0.278 eV/atom, indicating that the prediction performance of the model has been improved with the selection of key features and the elimination of redundant features. And compared with the relevant ABX_3_ perovskite stability work of Zhu et al. [5], our R^2^ of 0.94 is higher than theirs, 0.91, using the same algorithm. From this, we can see that the nine most important features are HF_X, PA_EN_AX, PA_EN_BX, GP_A, N_VE_B, IR_X, HF_B, SumDens, SumGP and SumN_VS (standing for fusion-heat of X-site element, Pauling electronegativity of A-site and B-site, the A-site group, the number of valence electrons at the B-site, the X-site ion radius, the heat of fusion at the B-site, the combined density, the combined group, and the combined number of p valence electrons; all the features and corresponding physical meanings used in this work are illustrated in Table S2, Supplementary Materials), among which HF_X is positively correlated, and PA_EN_AX and PA_EN_BX are negatively correlated. This can also be confirmed by the results of the Pearson correlation heat map in Figure 5b.

### 3.4. Model Validation

**Figure 6:**
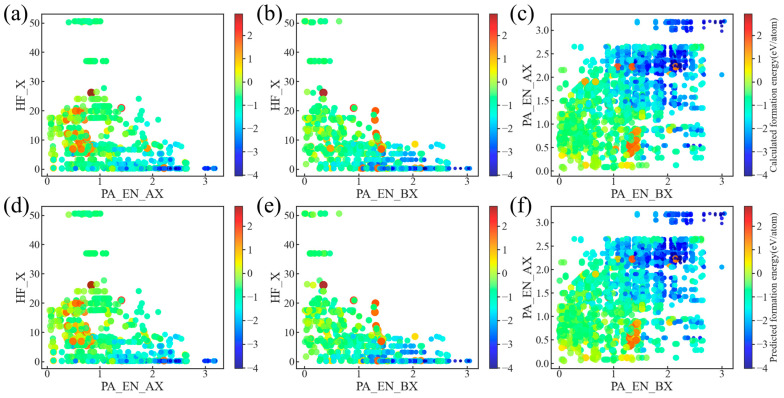
Distribution diagram of true value and ML-predicted value of formation energy for different features in 2D coordinates: (**a**–**c**) formation energy from Materials Project; (**d**–**f**) predicted formation energy obtained using XGBoost algorithm.

**Figure 7:**
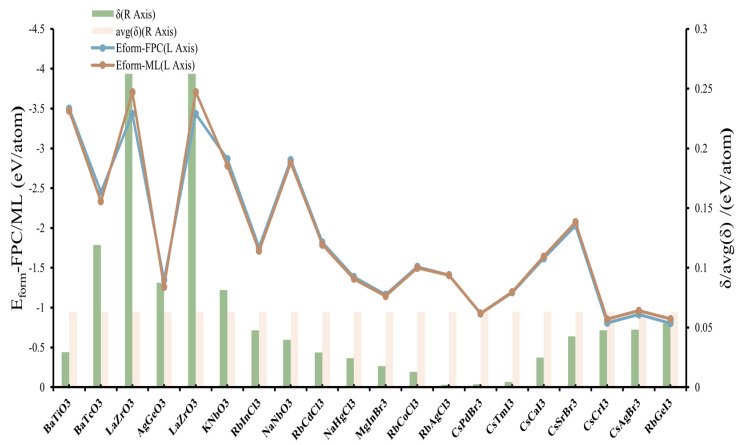
Comparison between ML-predicted results and FPC results.

In order to present the prediction results of our model for E_form_ more intuitively and evaluate it based on the traditional model evaluation criteria in the above work, we further validate the model. Figure 6 exhibits the comparison between the E_form_ distribution of compounds acquired randomly from the Materials Project database and the corresponding prediction results generated with our model. It can be seen that there is no significant difference between the two sets of distributions, indicating that our model has a high prediction accuracy. We used the acquired data to check the distribution of the nine features to verify whether the correlation between the features and E_form_ is consistent with our conjecture, as shown in Figure S2 (Supplementary Materials). Obviously, all nine features showed a high correlation with E_form_, and the trends are consistent with our conjecture, especially PA_EN_AX and PA_EN_BX, which showed high aggregation and clear negative relevance. We also randomly generated some compounds, performed FPC and model predictions simultaneously, and compared the results obtained (see Figure 7; detailed results in Table S1, Supplementary Materials).

## 4. Conclusions

In summary, to assess the instability of ABX_3_-type perovskites, we used ML methods to build a model to predict the formation energy of ABX_3_-type perovskites to guide the design of the perovskite structure. To save computing resources, we simplified the model while retaining the desired reliability and accuracy. Through multiple rounds of screening, our ML model features were gradually reduced from 168 per compound to only 9, achieving a goodness of fit R^2^ of 0.939 and a root mean square error of 0.278 eV/atom. We further verified the actual working of the model by comparing the FPC results with the model prediction results of random ABX_3_-type perovskite compounds. Our results showed that 75% of the values were correctly predicted within an error lower than 0.06. The model proposed here provides a powerful tool for the materials science community to accelerate the discovery and design of perovskite materials with minimized burden on computation.

## Figures and Tables

**Table 1:** Thirty input features selected and evaluated using the training results of various machine learning models.

Model	MAE (eV/atom)	RMSE (eV/atom)	MSE	R^2^	Pearson’s R Value	RMSE/Average ($y_{i}^{pred}$)
LR	0.473	0.607	0.369	0.706	0.843	0.353
SVR	0.229	0.375	0.140	0.888	0.942	0.221
MLP	0.221	0.352	0.124	0.901	0.950	0.214
DTR	0.229	0.407	0.165	0.868	0.934	0.235
RF	0.194	0.313	0.098	0.922	0.961	0.188
XGBoost	0.186	0.301	0.090	0.928	0.963	0.175

## Data Availability

The original contributions presented in this study are included in the article/Supplementary Material. Further inquiries can be directed to the corresponding author.

## Supplementary Materials

The following supporting information can be downloaded at https://www.mdpi.com/article/10.3390/ma18132927/s1. Figure S1. The probability distribution of the top 30 input features in the order of Pearson correlation importance from large to small. The 30 selected descriptors, whether they are continuous or scattered, are evenly distributed within their respective intervals, with no obvious anomalies, which means our descriptors are good; Figure S2. The scatter density distribution of the formation energy and 9 input features of the XGBoost model: (a–c) HF_X, PA_EN_AX, PA_EN_BX; (d–f) GP_A, N_VE_B, IR_X; (g–i) HF_B, SumDens, SumGP; Table S1. Eform calculated by FPC and predicted by ML model and differences in the 20 generated compounds; Table S2. Initial features and corresponding physical meanings; Table S3. Necessary software and packages and their version.
