# Supplementary material for: Prediction of ABX3 Perovskite Formation Energy Using Machine Learning
Source: Materials (Basel). 2025 Jun 20;18(13):2927. doi: 10.3390/ma18132927 (PMC12250765; doi:10.3390/ma18132927)
Supplement: Supplementary file 1 [file materials-18-02927-s001.zip › materials-3634683-supplementary.pdf]

Supplementary notes

for

Prediction of ABX3 Perovskite Formation Energy Using Machine Learning

1. **Supplementary note A:** the probability distribution of the 30 remaining features that indicated the relevance of the features.

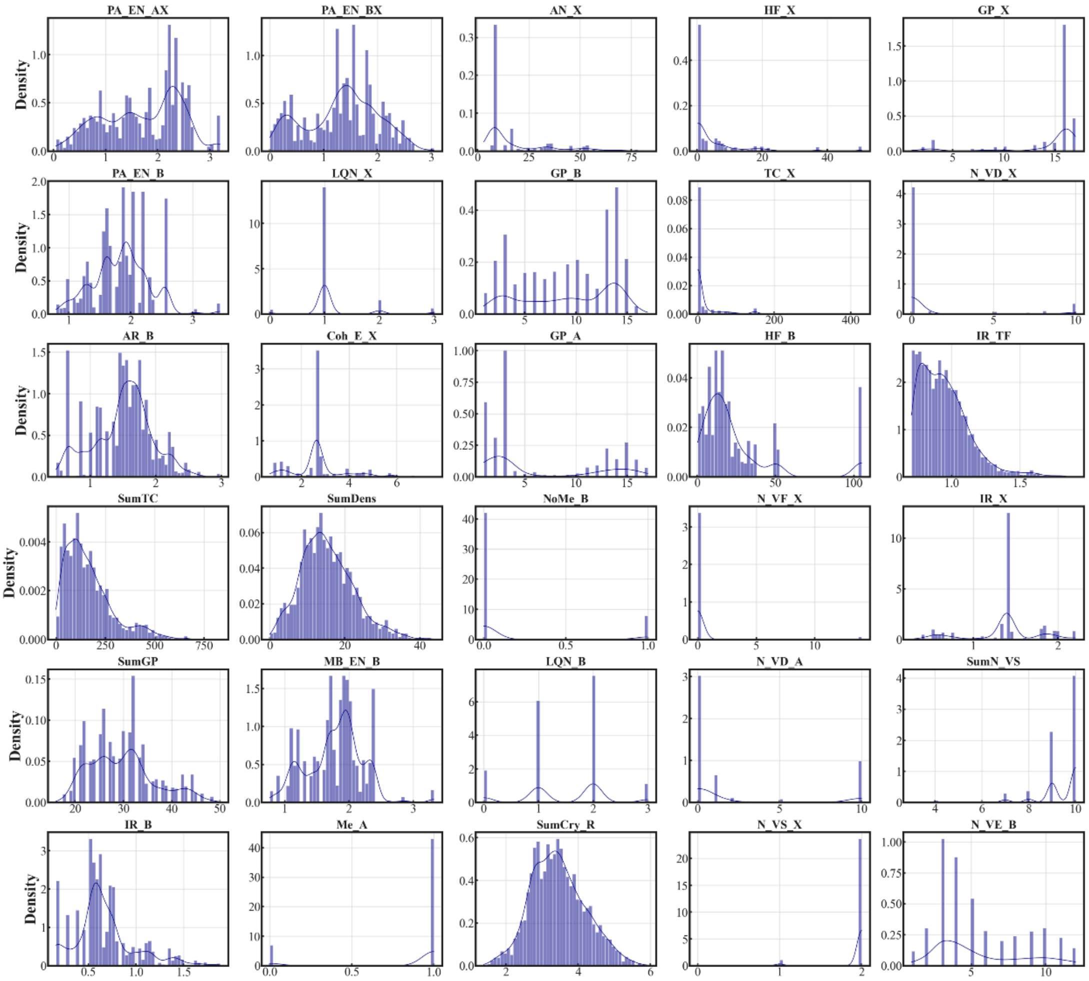

Figure S1. The probability distribution of the top 30 input features in the order of Pearson correlation importance from large to small. The 30 selected descriptors whether they are continuous or scattered, are evenly distributed within their respective intervals, with no obvious anomalies, which means our descriptors are good.

2. **Supplementary note B:** the scatter density distribution of the actual formation energy from our database towards the top 9 features, which indicated the aggregation and the trend of each feature, as well as the relevance.

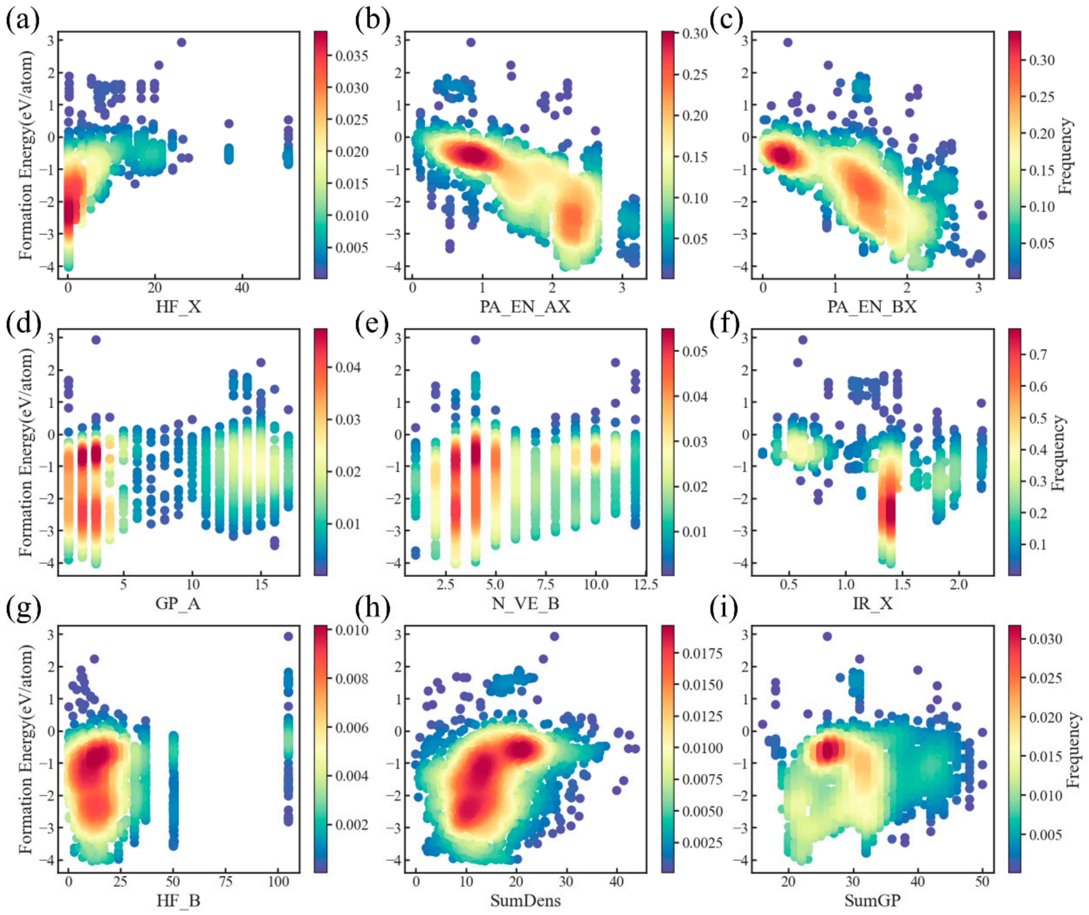

Figure S2. The scatter density distribution of the formation energy and 9 input features of XGBoost model:(a-c) HF_X, PA_EN_AX, PA_EN_BX; (d-f) GP_A, N_VE_B, IR_X; (g-i) HF_B, SumDens, SumGP.

3. **Supplementary note C:** the randomly generated 20 components and their formation energy performed by FPC and model predictions, as well as the difference of both ways.

Table S1. E_{form} calculated by FPC and predicted by ML model and differences of the 20 generated compounds.

Formula	E_{form}-FPC	E_{form}-ML	δ
**$BaTiO_3$**	-3.501502967	-3.472201109	0.029301858
**$BaTcO_3$**	-2.451314162	-2.332139015	0.119175147
**$LaZrO_3$**	-3.433824608	-3.706707954	0.272883347
**$AgGeO_3$**	-1.347726472	-1.260350466	0.087376007
**$LaZrO_3$**	-3.433824608	-3.706707954	0.272883347
**$KNbO_3$**	-2.865498339	-2.784202576	0.081295764
**$RbInCl_3$**	-1.75894174	-1.711284995	0.047656745
**$NaNbO_3$**	-2.856714312	-2.816859484	0.039854828
**RbCdCl3**	-1.81923984	-1.790038586	0.029201254
**$NaHgCl_3$**	-1.385024958	-1.360741854	0.024283105
**$MgInBr_3$**	-1.162705751	-1.145002007	0.017703743
**$RbCoCl_3$**	-1.512298307	-1.499384165	0.012914143
**$RbAgCl_3$**	-1.409284216	-1.407280564	0.002003652
**$CsPdBr_3$**	-0.925290382	-0.927815616	0.002525235
**$CsTmI_3$**	-1.191060319	-1.19563961	0.004579291
**$CsCaI_3$**	-1.617346192	-1.642319918	0.024973726
**$CsSrBr_3$**	-2.030199916	-2.07292223	0.042722313
**$CsCrI_3$**	-0.80728014	-0.855021477	0.047741337
**$CsAgBr_3$**	-0.913713074	-0.961898983	0.048185908
**$RbGeI_3$**	-0.803979863	-0.857450247	0.053470384

4. **Supplementary note D:** the features used in this work and their corresponding physical meanings, for any feature with A/B/X behind them e.g. ANA/B/X, stands for the A/B/X-Site feature and their property.

Table S2. Initial features and corresponding physical meaning.

No.	Features	Physical meaning
1	AN	Atomic Number
2	AW	Atomic Weight
3	Per	Period
4	GP	Group
5	Fam	Families
6	Me	Metal
7	NoMe	Nonmetal
8	Mell	Metalloid
9	MN	Mendeleev Number
10	LQN	L quantum number
11	AR	Atomic radius
12	Cov_R	Covalent Radius
13	ZRS	Zunger radii sum
14	IR	Ionic radius
15	Cry_R	Crystal radius（Å），
16	PA_EN	Pauling electronegativity
17	MB_EN	Martynov & Batsanov electronegativity
18	AR_EN	Allred-Rockow electronegativity
19	N_VE	Number of valence electrons
20	N_VS	Number of s valence electrons
21	N_VP	Number of p valence electrons
22	N_VD	Number of d valence electrons
23	N_VF	Number of f valence electrons
24	UN_VS	Unfilled s valence electrons
25	UN_VP	Unfilled p valence electrons
26	UN_VD	Unfilled d valence electrons
27	UN_VF	Unfilled f valence electrons
28	N_OSE	Outer shell electrons
29	1st_IP	1st ionization potential
30	P	Polarizability
31	MP	Melting point
32	BP	Boiling point
33	Dens	Density
34	SH	Specific heat

35	HF	Heat of fusion
36	HV	Heat of vaporization
37	TC	Thermal conductivity
38	Coh_E	Cohesive energy
39	Form_EN	Formation energy

5. **Supplementary note E:** the necessary software and packages for this work, as well as their version.

Table S3. Necessary software and packages and their version.

No.	Name	Version
1	Python	3.7.16
2	Jupyter	1.0.0
3	Qmpy	1.4.0
4	Seaborn	0.12.2
5	Shap	0.41.0
6	Toml	0.10.2
7	Xgboost	1.6.2
8	Numpy	1.21.6
9	Matminer	0.8.0
10	Matplotlib-base	3.2.2
11	Matplotlib-inline	0.1.6
12	Notebook	6.5.2
13	Openpyxl	3.1.2
14	Openssl	1.1.1
15	Pandas	1.3.5
16	Ipykernel	6.15.2
17	Ipython	7.31.1
18	pip	22.3.1
19	Plotly	5.14.1
20	Pyqt	5.15.7
21	pyqt5-sip	12.11.0
22	qmpy-rester	0.2.0
23	json5	0.9.6
24	Scikit-learn	1.0.2
25	Pygments	2.11.2
26	Pymatgen	2022.1.7
